# Postcolonoscopy Colorectal Cancer as a Quality Metric—Identifying Opportunities to Improve Outcomes

**DOI:** 10.1001/jamanetworkopen.2023.6680

**Published:** 2023-04-03

**Authors:** Abraham Segura, Shivan J. Mehta

**Affiliations:** Division of Gastroenterology, Department of Medicine, University of Pennsylvania Perelman School of Medicine, Philadelphia; Division of Gastroenterology, Department of Medicine, University of Pennsylvania Perelman School of Medicine, Philadelphia; Center for Health Care Innovation, University of Pennsylvania, Philadelphia

Colorectal cancer (CRC) remains one of the most common types of cancer diagnosed and a leading cause of death worldwide.^1^ Colonoscopy is commonly performed for diagnostic and screening purposes and is the preferred screening test in the United States because of its capacity to reduce the risk of CRC incidence and mortality by early detection and removal of asymptomatic premalignant lesions. It is, however, highly operator dependent and influenced by patient and process barriers, which may result in missed lesions. Physician adenoma detection rate (ADR) is inversely associated with interval CRC after colonoscopy and is recommended for use as a quality metric by specialty societies and as a reportable quality measure. However, ADR may not capture missed cancers due to inadequate polypectomy or in colonoscopy done for diagnostic or surveillance purposes.^2^

Postcolonoscopy CRC (PCCRC) is an alternative quality metric that tracks the rate of cancers occurring after a normal colonoscopy result. Prior studies estimate that PCCRC accounts for 2% to 8% of all CRCs, and most of these cases are attributed to missed or incompletely resected lesions.^3^ The use of PCCRC as a benchmark in practice, however, has been limited by relative infrequency of the outcome and inconsistent definition of interval lengths following colonoscopy. To address these limitations, the World Endoscopic Organization published guidelines recommending reporting the PCCRC rate for an interval of 3 years (PCCRC-3y) to establish a reliable gauge for evaluating and comparing health systems.^4^

In *JAMA Network Open*, Kahi and colleagues^5^ evaluated the prevalence and mortality of patients with PCCRC-3y receiving care in the Veterans Health Administration (VA). They performed a retrospective cohort study using VA-Medicare administrative data that identified a total of 29 877 incident cases of CRC between 2003 and 2013, of which 1785 (6.0%) were classified as PCCRC-3y. Primary outcome analysis revealed that patients with PCCRC-3y had similar rates of all-cause and cause-specific mortality compared with those with detected CRC within 6 months of colonoscopy (adjusted hazard ratios [aHRs], 1.04 [95% CI, 0.98–1.11] and 1.04 [95% CI, 0.95–1.13], respectively). Notably, patients without any colonoscopy within 3 years (no colonoscopy) of CRC diagnosis had significantly higher all-cause (aHR 1.76, 95% CI 1.70–1.82) and cause-specific (aHR 2.22, 95% CI 2.12–2.32) mortality compared with the PCCRC-3y group. Nearly 93% of patients in the no colonoscopy group had a colonoscopy performed more than 10 years before CRC diagnosis or did not have a colonoscopy documented in the medical record. Secondary analyses showed that patients with PCCRC-3y were more likely to present with bowel obstruction or peritonitis, more likely to have index colonoscopy outside the VA health system, and less likely to have their colonoscopy performed by a gastroenterology specialist.

The authors should be commended for studying a relevant question with clinical and policy implications, adopting a rigorous, standardized definition for PCCRC, and evaluating robust cancer mortality outcomes in a large integrated health care system in the US. Prior cohort studies that have evaluated PCCRC have incorporated one, but not all, of these strengths. This study also has limitations that merit consideration. First, the authors mention they were unable to measure other quality metrics, including ADR. This omission does not necessarily detract from the outcomes presented, but it is a missed opportunity to evaluate how well ADR correlates with PCCRC-3y in the VA health care system. Prior research reports that colonoscopies performed in the VA setting have significantly higher ADRs than those done outside the VA setting; yet this study suggests PCCRC rates are comparable between these settings.^6^ Second, generalizability may be limited given the predominantly male, White population studied. We agree with the authors that the consistency in reported PCCRC rates with those from prior studies suggests internal validity is preserved and that results from this study may translate into other settings. Third, we note that the influence of race and ethnicity on PCCRC detection and outcomes is not fully explored. Prior research in Medicare beneficiaries has identified racial and ethnic differences in interval CRC.^7^ In this study, however, there is no similar analysis that explicitly evaluates PCCRC prevalence and mortality outcomes by race and ethnicity, although they did adjust for it as covariates in the multivariable models. These results may provide valuable insight and potentially identify health care disparities meriting further study. Additionally, it may be interesting to evaluate colonoscopy indication and type of anesthesia received, as these may impact PCCRC.

The results from this study provide insight into the quality of care within the VA health care system and provide a framework for using PCCRC as a quality metric on a system level. The authors suggest that colonoscopy quality is comparable between the VA and other health care systems and indicate that patients who receive care outside the VA setting or from nongastroenterologists may face increased risk of PCCRC. This difference in PCCRC may be explained by differential ascertainment of data, a reduced focus on colonoscopy quality outside the VA system, or operator differences. This may have important policy implications on the recently expanded Veterans Choice Act, which extends coverage to care outside the VA system for veterans with access limited by distance, transportation, burden of disease, or internal delays in care. Although all-cause and cancer-specific mortality were similar between patients with colonoscopies that missed lesions vs those with colonoscopies that detected CRC, patients classified with PCCRC-3y were more likely to present with complications requiring unplanned hospitalization. More importantly, patients without any colonoscopy had significantly higher rates of all-cause and cancer-specific mortality, which underscores the importance of regular screening and access to diagnostic testing in symptomatic individuals. High-quality colonoscopy is certainly important, though it is of limited value without patients to treat. Access and adherence to colonoscopy remains a significant barrier in CRC care, which disproportionately burdens minoritized racial and ethnic groups. Additional efforts must continue to identify modifiable risk factors and study culturally appropriate interventions that increase adherence.

In summary, Kahi et al^5^ demonstrate that colonoscopy quality in the VA system is comparable to that of other health settings using PCCRC-3y as a benchmark, and that patients classified with PCCRC-3y have similar mortality event rates to those with CRC detected by colonoscopy, but lower mortality compared to those without colonoscopy. Further research to evaluate the influence of race and ethnicity, differential care settings, and other established quality metrics on PCCRC-3y will be important. Colorectal cancer screening adherence with colonoscopy remains a protective factor, and efforts to expand its reach remain a larger opportunity space.
